# A nomogram based on ultrasonographic features and clinical indicators for differentiating mass-forming intrahepatic cholangiocarcinoma and liver metastatic colorectal adenocarcinoma

**DOI:** 10.3389/fonc.2023.1245686

**Published:** 2023-10-31

**Authors:** Wuyongga Bao, Min Liao, Jie Yang, Jiayan Huang, Keyu Zeng, Qiang Lu

**Affiliations:** Department of Medical Ultrasound, West China Hospital, Sichuan University, Chengdu, Sichuan, China

**Keywords:** mass-forming intrahepatic cholangiocarcinoma, metastatic colorectal adenocarcinoma, contrast-enhanced ultrasound, nomogram, predictive model

## Abstract

**Objective:**

This study aimed to develop and validate a nomogram based on ultrasonographic features and clinical indicators to differentiate mass-forming intrahepatic cholangiocarcinoma (MF-ICC) from hepatic metastatic colorectal adenocarcinoma.

**Materials and methods:**

A total of 343 patients with pathologically confirmed MF-ICC or metastatic colorectal adenocarcinoma were enrolled between October 2018 and July 2022. Patients were randomly assigned to training and validation sets at a ratio of 7:3. Preoperative ultrasound features and clinical indicators were retrieved. Univariate logistic regression analysis was employed to select relevant features. Multivariate logistic regression analysis was used to establish a predictive model, which was presented as a nomogram in training sets. The model’s performance was assessed in terms of discrimination, calibration, and clinical usefulness.

**Results:**

The study included 169 patients with MF-ICC and 174 with liver metastatic colorectal adenocarcinoma, assigned to training (n=238) and validation (n=105) cohorts. The nomogram incorporated ultrasound features (tumor size, lesion number, echogenicity, tumor necrosis, and rim arterial phase hyperenhancement) and clinical information (serum levels of CEA, CA19-9, CA125). The nomogram demonstrated promising performance in differentiating these two entities in both training and validation sets, with an AUC value of 0.937 (95%CI: 0.907,0.969) and 0.916 (95%CI: 0.863,0.968), respectively. The Hosmer–Lemeshow test and calibration curves confirmed good consistency between predictions and observations. Additionally, decision curve analysis confirmed the nomogram’s high clinical practicability.

**Conclusion:**

The nomogram based on ultrasound features and clinical indicators demonstrated good discrimination performance in differentiating MF-ICC from metastatic colorectal adenocarcinoma, which may enhance clinical decision-making process in managing these challenging diagnostic scenarios.

## 1 Introduction

Intrahepatic cholangiocarcinoma (ICC), comprising 10-15% of all primary liver cancer cases, is the second most common liver cancer after hepatocellular carcinoma (HCC). Its incidence and mortality have been increasing during the last decade (1, 2), and patients with ICC often remain asymptomatic in the early stages, leading to delayed diagnosis and poor clinical outcomes (3, 4). Surgical resection remains the primary curative treatment option for ICC (5); however, delayed diagnosis often precludes effective surgical intervention.

ICC frequently arises in the noncirrhotic liver (6, 7), and its diverse clinical presentations may pose diagnostic challenges for even experienced radiologists. Although various contrast-enhanced ultrasound (CEUS) patterns have been described for MF-ICC (mass-forming intrahepatic cholangiocarcinoma) (8, 9), liver metastatic adenocarcinoma, particularly from gastrointestinal system, can exhibit similar patterns (10, 11). In addition, as both types of tumors exhibit adenocarcinoma histology, it is sometimes hard to differentiate based on the histological or imaging analysis. Given that treatment strategies for these two diseases differ substantially, differentiation between MF-ICC and metastases of adenocarcinoma is critical for optimal patient management.

To the best of our knowledge, there is a paucity of literature focusing on distinguishing between MF-ICC and metastatic adenocarcinoma from gastrointestinal organs utilizing ultrasound features and clinical indicators. Therefore, the aim of our study was to develop and validate a nomogram incorporating clinical indicators, B-mode ultrasound (BMUS) features, and CEUS characteristics to differentiate between MF-ICC and metastatic colorectal adenocarcinoma, with the goal of improving clinical decision-making and patient outcomes.

## 2 Materials and methods

### 2.1 Patient selection

**Figure 1:**
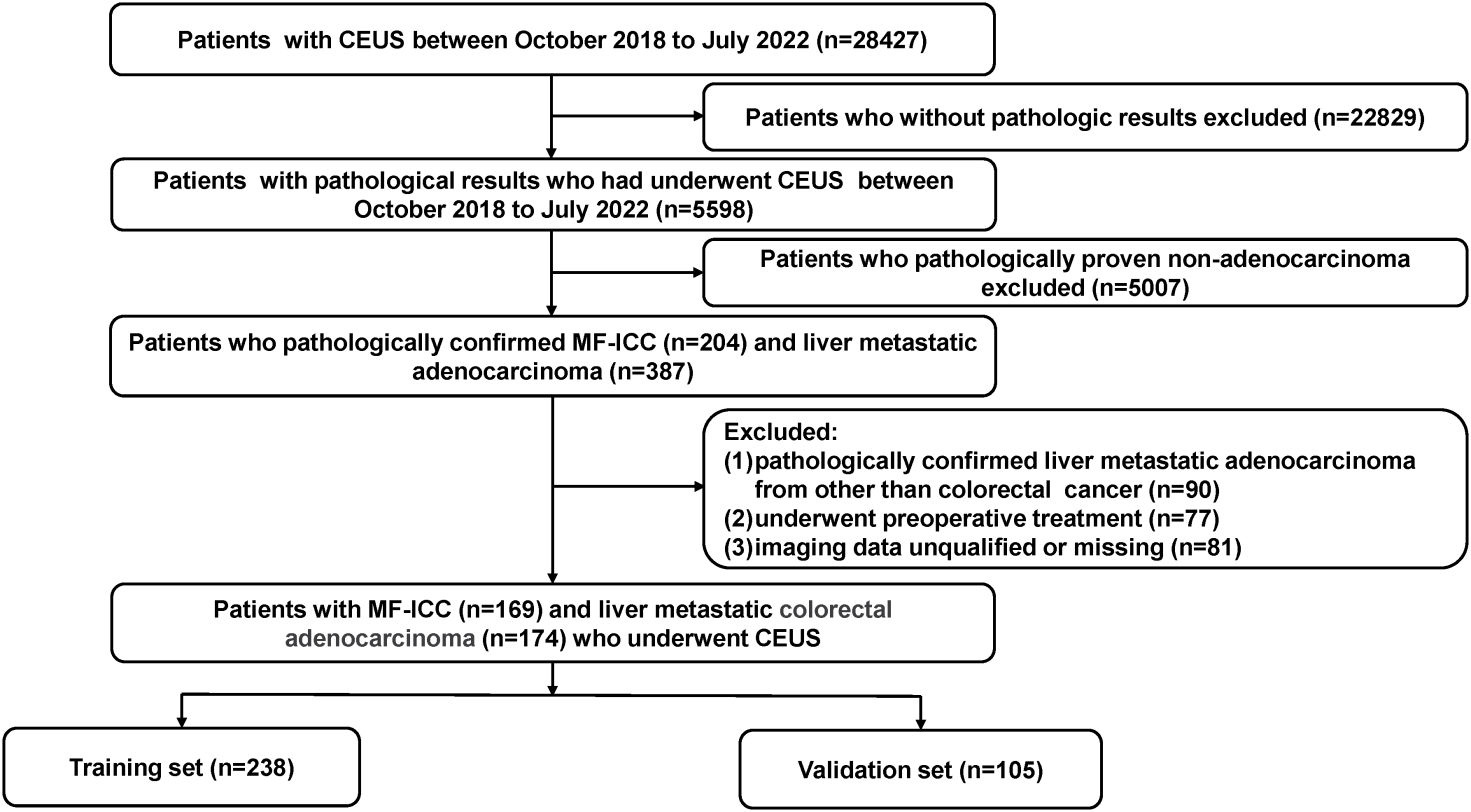
Flow chart of the patient selection process. MF-ICC, mass-forming intrahepatic cholangiocarcinoma; CEUS, contrast enhanced ultrasound.

Approval for this retrospective study was granted by the institutional research ethics review board. Informed consent from patients was deemed unnecessary and waived. From October 2018 and July 2022, consecutive participants with pathologically proven MF-ICC and liver metastatic adenocarcinoma were enrolled in our study. Patients who had undergone CEUS examination within 1 month prior to biopsy or surgical resection were included. Exclusion criteria were as follows: 1. Liver metastatic adenocarcinoma originating from sources other than colorectal cancer; 2. Patients who had received preoperative anticancer treatment (chemotherapy, radiotherapy, or targeted therapy); 3. Patients with incomplete imaging data. Finally, a total of 343 patients were included and randomly divided into two groups, the training cohort (n=238) and the validation cohort (n=105), as shown in **Figure 1**. Baseline clinical data, including age, gender, liver hepatitis, and serum tumor marker levels such as alpha fetoprotein (AFP), carbohydrate antigen 19-9 (CA19-9), carcinoembryonic antigen (CEA), and carbohydrate antigen 125 (CA125), were obtained from medical records.

### 2.2 Ultrasound imaging acquisition

BMUS and CEUS examinations were performed by using Philips IU 22 or Mindray Resona 7 ultrasound system equipped with a C5-1 or SC6-1U abdominal convex probe. A dose of 1.2~2.4 ml of SonoVue (Bracco, Milan, Italy) was injected and immediately followed by 5 ml of 0.9% sodium chloride solution. The imaging timer was initiated simultaneously post-injection. The set of CEUS imaging was digitally stored on the hard disk of the ultrasound system for subsequent analysis.

### 2.3 Ultrasound image assessment

The largest lesion was selected for patients with multiple liver lesions. BMUS and CEUS images were blindly reviewed by two experienced radiologists to assess the characteristics of the lesions.

The BMUS features evaluated included echogenicity, shape, and boundary of the tumor. The CEUS features assessed included patterns of arterial phase hyperenhancement (APHE), tumor necrosis, necrosis area, early washout, marked washout, and unclear boundary of intratumor non-enhanced area. Abnormal lymph nodes, intrahepatic bile duct dilation, bile duct stone, maximal diameter of the targeted nodule, and number of lesions were also extracted from the ultrasound reports. The hyperenhancement of the lesion in the arterial phase was classified as rim or not rim, while the washout time was divided into <60s or ≥60s. Additionally, the necrosis area was categorized into three groups: absence, <50%, and ≥50%. The unclear boundary of intratumor non-enhanced area referred to an obscure defect within the hypo-enhancement area in the portal venous or late phase with an unclear boundary (12).

### 2.4 Model construction and validation

A predictive model for differentiating between MF-ICCs and metastatic colorectal adenocarcinoma was developed using multivariate logistic regression analysis with BMUS, CEUS, and clinical features. In the chi-square test, when the p-value of a variable is less than 0.01, that variable is selected to be included in further univariate logistic regression analysis. The variables that achieved a significance level of P<0.01 in the univariate analysis were included in the multivariate logistic regression analysis. To select the most significant predictive features among all the clinical indicators and ultrasonographic characteristics in the training cohort, multicollinearity was assessed by calculating variation inflation factors and condition indexes. Based on the selected variables, a nomogram was formulated. Internal validation was conducted to determine the diagnostic performance of the predictive model. Discrimination ability was measured using the concordance index (C-index), which ranges from 0.5 to 1.0, with 0.5 indicating no predictive effect and 1.0 indicating complete concordance between predicted and actual results. The distinguishability of the nomogram was estimated using receiver operating characteristic (ROC) curve analysis. Calibration of the nomogram was assessed using the Hosmer-Lemeshow test and the calibration curve. The predictive performance of the nomogram was evaluated using decision curve analysis (DCA).

### 2.5 Statistical analysis

The patients were randomly allocated to training and validation sets at a ratio of 7:3 using SPSS version 19.0 software (IBM Corporation, Armonk, NY). The quantitative data were expressed as means ± standard deviations, while the qualitative data were presented as absolute numbers and percentages. The cut-off values for AFP, CEA, CA125, and CA19-9 were set at 20 ng/mL, 5 ng/mL, 35 ng/mL, and 36 U/mL, respectively, based on previous studies (13, 14). The comparison of clinical and imaging features between the MF-ICC and metastases groups was performed using independent sample t-test, Pearson chi-square test, or Fisher’s exact test. Interobserver agreement between the two radiologists was evaluated using kappa (k) statistics. Logistic regression, nomogram generation, ROC curve analysis, C-index calculation, calibration curve generation, Hosmer-Lemeshow test, and other statistical analyses were conducted using Stata version 16.0 (Stata Corp, College Station, TX). Statistical significance was set at P<0.05.

## 3 Results

### 3.1 Patient characteristics

A total of 343 patients were enrolled in this study, comprising 169 individuals with MF-ICC and 174 patients with liver metastatic colorectal adenocarcinoma. Patients were randomly allocated to training sets (n=238) and validation sets (n=105). The clinical characteristics of the patients were compared, and the results were presented in **Table 1**. Significant differences were observed in the serum level of AFP (P=0.005, P=0.01, respectively), CA19-9 (P<0.001, P<0.001, respectively), CEA (P<0.001, P=0.019, respectively), CA125 (P<0.001, P=0.007, respectively) in both training and validation sets.

**Table 1:** Clinical characteristics of MF-ICC and liver metastatic colorectal adenocarcinoma in training and validation sets.

Variables	Training set (n=238)		*P* value	Validation set (n=105)		*P* value
	MF-ICC	Liver metastasis		MF-ICC	Liver metastasis	
**Age(years)**	59.65 ± 10.44	58.73 ± 10.67	0.67	59.61 ± 10.24	60.89 ± 11.99	0.59
**Gender(male/female)**	69/49	84/36	0.064	27/24	38/16	0.066
**Hepatitis status**			0.012			0.874
HBV (+)	25(22.12)	10(8.85)		7(14.29)	7(13.21)	
HCV (+)	1(0.88)	0 (0)		0(0)	0(0)	
Others	87(76.99)	103(91.15)		42(85.71)	46(86.79)	
**AFP (**≥20**ng/mL)**	8(6.78)	0(0)	0.005	6(12.00)	0(0)	0.01
**CA19-9 (**≥36**U/mL)**	72(63.72)	37(32.17)	<0.001	30(58.82)	12(22.22)	<0.001
**CEA (**≥5**ng/mL)**	30(26.09)	77(65.25)	<0.001	15(29.41)	28(51.85)	0.019
**CA125 (**≥35**ng/mL)**	36(33.96)	7(6.48)	<0.001	16(32.65)	5(10.20)	0.007

Data are presented as means ± standard deviations and the number (percentage).

MF-ICC, mass-forming intrahepatic cholangiocarcinoma; AFP, alpha fetoprotein; CA19-9, carbohydrate antigen 19-9; CEA, carcinoembryonic antigen; CA125, carbohydrate antigen 125.

### 3.2 BMUS and CEUS characteristics

A comparison of the imaging features of MF-ICC and metastatic colorectal adenocarcinoma were presented in **Table 2**. In both the training and validation sets, the frequency of tumor size ≥5cm, abnormal lymph node, bile duct dilation, and hypoechoic features and irregular shape were higher in the MF-ICC group than in the metastasis group on BMUS. Single lesion was more commonly observed in the MF-ICC group than in the metastasis group in the training sets (P=0.002). Regarding CEUS features, rim APHE (P=0.001, P=0.04, respectively) was more frequently detected in the metastasis group in both the training and validation sets. Unclear intratumoral boundaries, tumor necrosis, and necrosis areas ≥50% were more commonly observed in the MF-ICC group than in the metastasis group in both training and validation sets. The interobserver agreement for the review of ultrasound features is shown in **Supplementary Table S1**, with kappa ranging from 0.095 to 0.694.

**Table 2:** BMUS and CEUS features of MF-ICC and liver metastatic colorectal adenocarcinoma in training and validation sets.

Variables	Training set (n=238)		*P* value	Validation set (n=105)		*P* value
	MF-ICC	Liver metastasis		MF-ICC	Liver metastasis	
BMUS features						
**Lesion size (**≥5**cm)**	44(37.29)	102(85)	<0.001	19(37.25)	48(85.19)	<0.001
**Lesion number(single)**	76(64.41)	53(44.17)	0.002	31(60.78)	29(53.70)	0.464
**Abnormal lymph node**	22(18.64)	0(0)	<0.001	5(9.80)	0(0)	0.018
**Bile duct dilation**	20(16.95)	2(1.67)	<0.001	12(23.53)	3(5.56)	0.009
**Bile duct stone**	4(3.39)	1(0.83)	0.169	2(3.92)	0(0)	0.142
**Echogenicity**			<0.001			<0.001
Hypo	102(86.44)	69(57.50)		48(94.12)	29(53.70)	
Iso	3(2.54)	8(6.67)		1(1.96)	3(5.56)	
Hyper	13(11.02)	43(35.83)		2(3.92)	22(40.74)	
**Ill-defined shape**	93(78.81)	71(59.17)	0.001	40(78.43)	32(59.26)	0.034
**Irregular boundary**	92(77.97)	81(67.50)	0.07	44(86.27)	34(62.96)	0.006
CEUS features						
**Rim APHE**	43(36.44)	70(58.33)	0.001	16(31.37)	27(50.00)	0.04
**Early washout**	53(44.92)	51(42.50)	0.707	30(58.82)	22(40.74)	0.08
**Marked washout**	15(12.71)	19(15.83)	0.579	10(19.61)	9(16.67)	0.802
**Unclear boundary of intratumor non-enhanced area**	24(20.34)	10(8.33)	0.008	13(25.49)	3(5.56)	0.005
**Tumor necrosis**	50(42.37)	25(20.83)	<0.001	26(50.98)	10(18.52)	<0.001
**Necrosis area**			0.002			0.002
Absent	68(57.63)	95(79.17)		25(49.02)	44(81.48)	
< 50% area	20(16.95)	9(7.50)		16(31.37)	6(11.11)	
≥ 50% area	30(25.42)	16(13.33)		10(19.61)	4(7.41)	

Data are presented as the number (percentage).

BMUS, B- mode ultrasound; CEUS, contrast enhanced ultrasound; MF-ICC, mass-forming intrahepatic cholangiocarcinoma; APHE, arterial phase hyper- enhancement.

### 3.3 Prediction model and nomogram construction and validation

**Figure 2:**
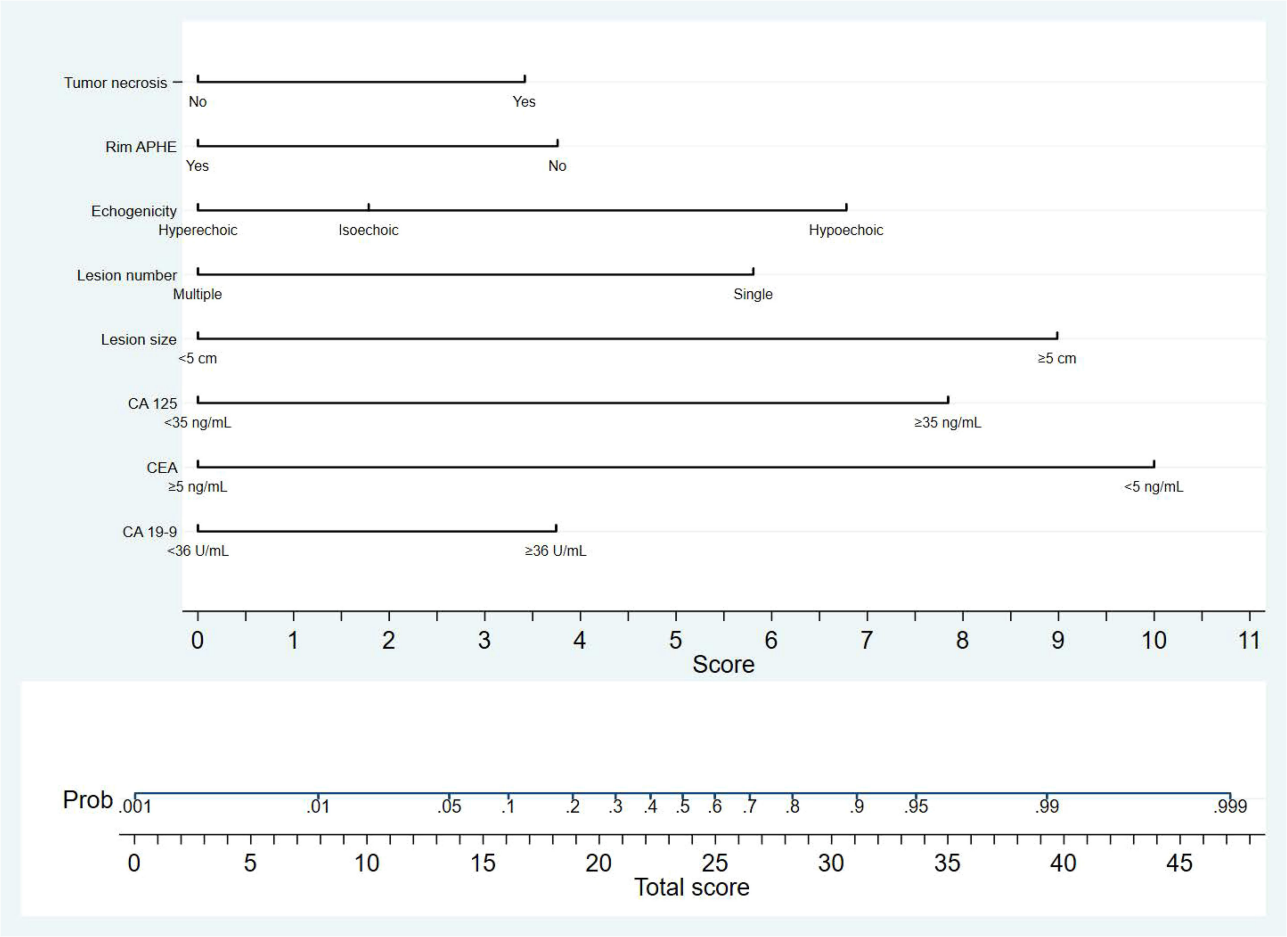
The nomogram developed in the study is a graphical tool that predicts the probability of MF-ICC based on specific sonographic features and clinical information. The variables included in the nomogram are single lesion, tumor size≥5cm, hypo-echogenicity, presence of tumor necrosis, absence of rim APHE, elevated serum level of CA-125, CA19-9, and lower CEA level. Each variable was assigned corresponding predictor points from the point scale, which was drawn at the top of the nomogram. The sum of the points of each variable is projected onto the bottom scale to determine the probability of MF-ICC.

**Figure 3:**
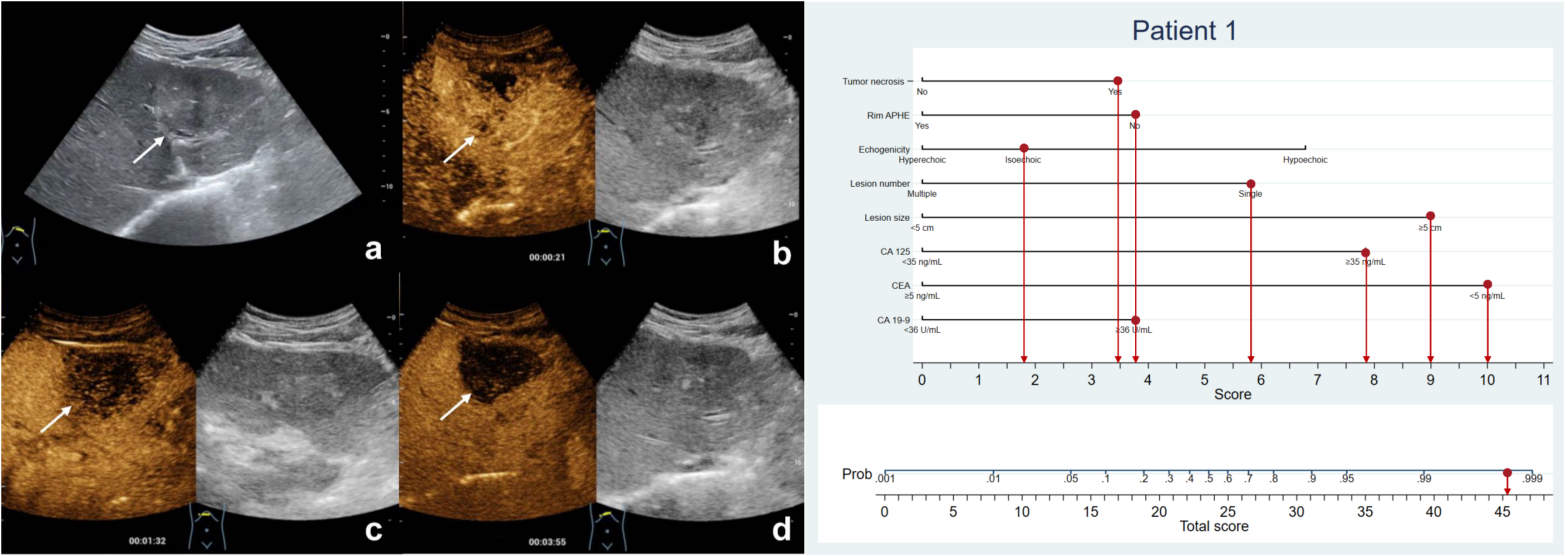
The patient was a 57-year-old man with a 6.1-cm isoechoic solid lesion in the liver **(A)**. Hetero-hyperenhancement was observed during the arterial phase **(B)**, and the lesion showed necrosis areas in the central on contrast-enhanced ultrasound **(B–D)**. The serum level of CA125 and CA19-9 were elevated, while CEA level was normal. Based on the nomogram, a total of 45.2 points were assigned to the patient, corresponding to a probability of more than 90% of having MF-ICC. Postoperative pathological examination confirmed the diagnosis of MF-ICC.

**Figure 4:**
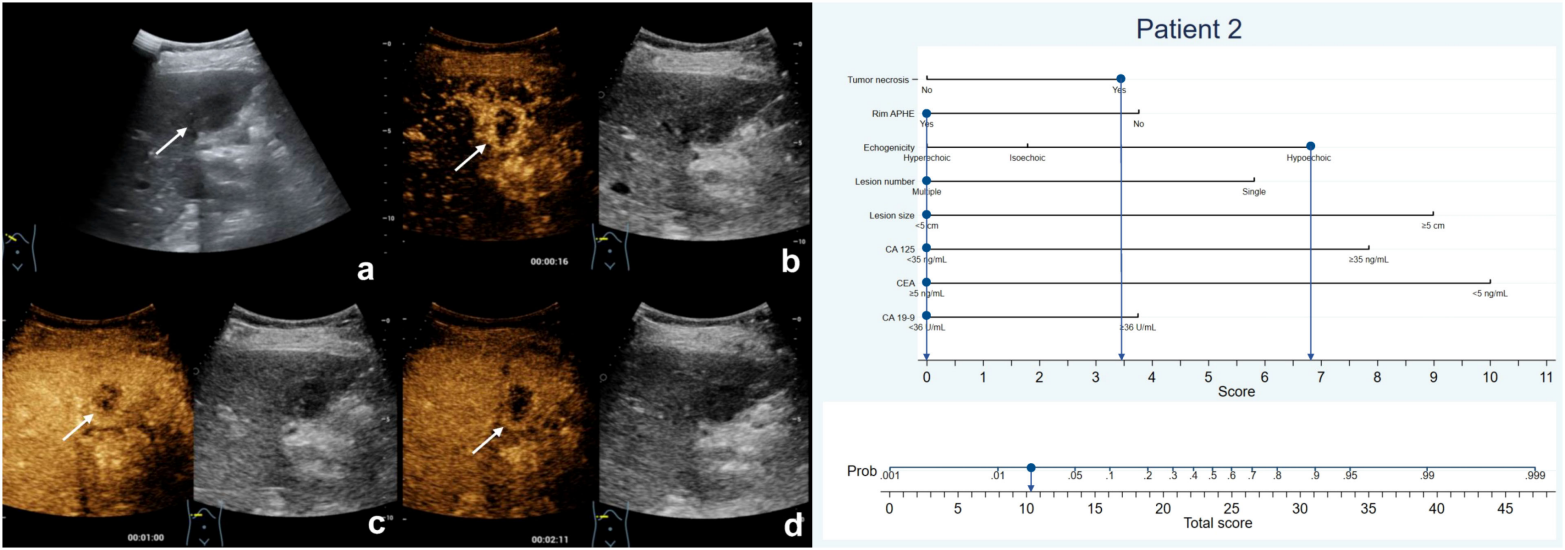
The patient was a 75-year-old female who presented with several hypoechoic lesions in liver, with the largest one measuring 2.5-cm in diameter **(A)**. Rim hyperenhancement of the tumor was observed during the arterial phase **(B)**, and contrast-enhanced ultrasound revealed necrotic areas within the tumor **(B–D)**. The patient exhibited elevated serum CEA levels but normal CA19-9 and CA125 levels. The nomogram score assigned to the patient was 10.2, indicating a less than 10% chance of developing MF-ICC. Pathological analysis confirmed liver metastatic adenocarcinoma originating from colon.

**Figure 5:**
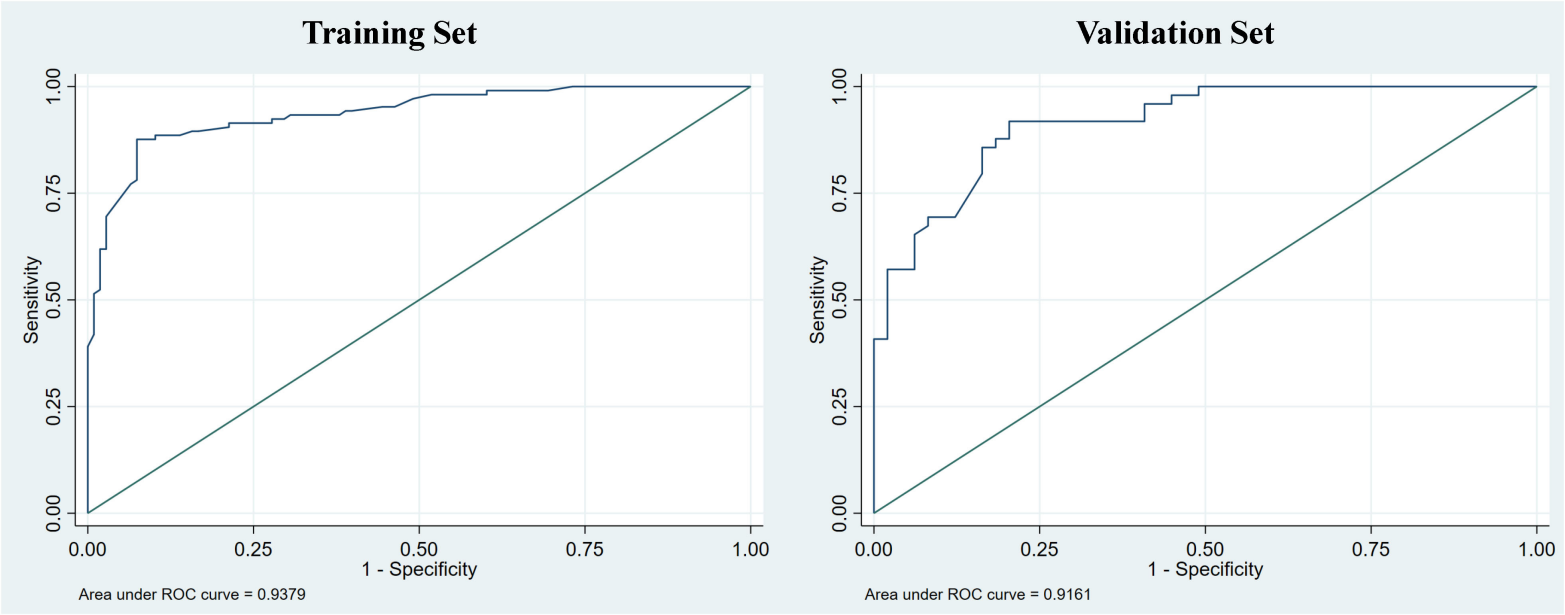
The receiver operating curve (ROC) of the nomogram was evaluated in both the training and validation sets, with corresponding area under the curve (AUC) values of 0.938 and 0.916, respectively.

**Figure 6:**
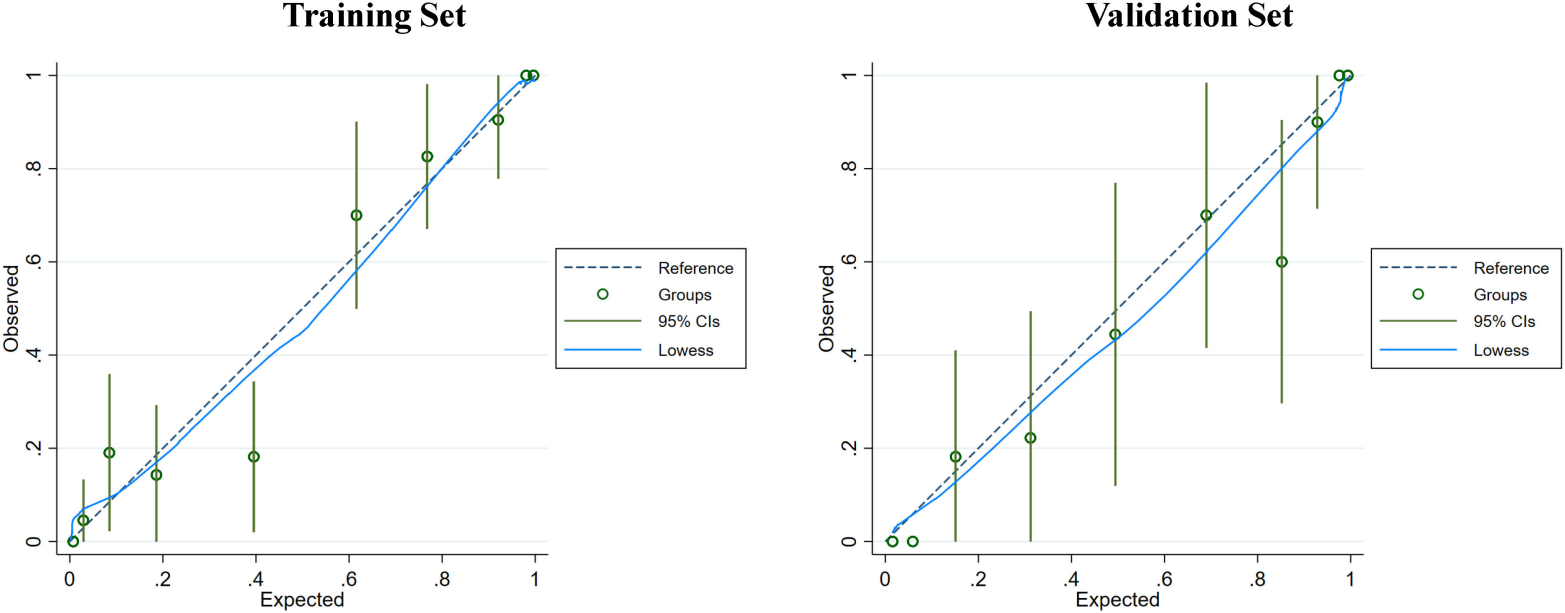
The calibration curve of the nomogram was assessed in both the training and validation sets to evaluate the consistency between the predicted probability of MF-ICC and the observed outcomes. The dashed line represents an ideal model with perfect prediction. The blue solid lines represent the performance of the nomogram in the training and validation sets, respectively. A closer fit to the diagonal dashed line indicates superior prediction accuracy. Furthermore, the Hosmer-Lemeshow test demonstrated p-values of 0.489 and 0.759 in the training and validation sets, respectively.

**Figure 7:**
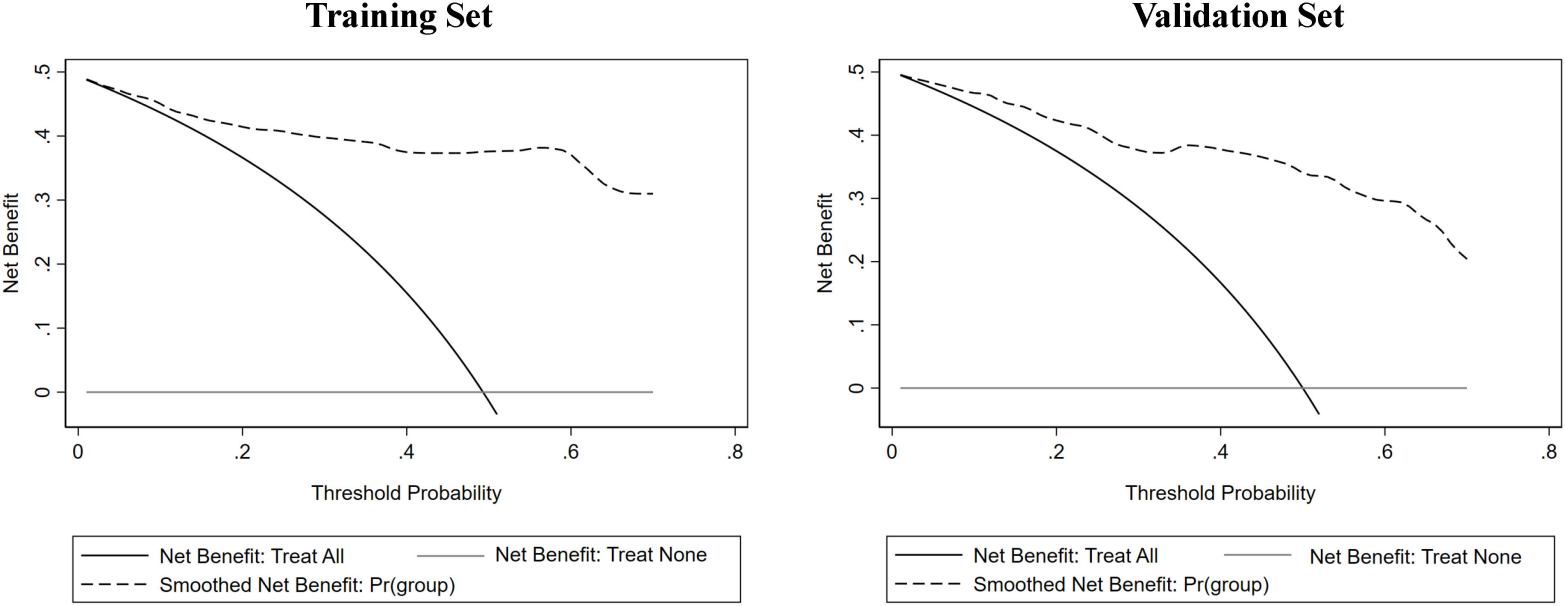
The decision curve analysis (DCA) of the nomogram was conducted on both the training and validation sets, revealing that the nomogram offered considerable clinical net benefit in comparison to both the treat-all-patients strategy (solid gray line) and the treat-none strategy (horizontal solid black line).

Clinical indicators and ultrasound features were extracted from the training sets for further multivariate logistic regression analysis when P < 0.01 using univariate logistic regression analysis. After assessing the multicollinearity among included variables, unclear intratumoral boundaries and necrosis area were omitted from the final multivariate analysis due to their collinear nature. The final selections included elevated CA19-9 (P < 0.001), CA-125 level (P < 0.001), normal CEA level (P < 0.001), tumor size ≥ 5cm (P < 0.001), single lesion (P=0.002), hypo-echogenicity (P < 0.001), irregular shape (P=0.001), tumor necrosis (P < 0.001), and rim APHE (P <0.001) for multivariable logistic regression analysis (**Table 3**). According to the multivariate logistic regression analysis, irregular shape (P=0.173) was not independent factors for the diagnosis of MF-ICC. The remaining variables were incorporated into the predictive model, and a nomogram was constructed (**Figures 2**–**4**). The final prediction nomogram exhibited high overall classification performance for differentiating MF-ICCs from metastatic colorectal adenocarcinoma, with an AUC value of 0.937 (95%CI: 0.907,0.969) in training sets and 0.916 (95%CI: 0.863,0.968) in validation sets (**Figure 5**). The calibration curve of the nomogram demonstrated good agreement between the predicted and actual outcomes of MF-ICC (**Figure 6**). The Hosmer-Lemeshow x2 in the training and validation set was 9.46 (P = 0.489) and 6.63 (P = 0.759), respectively. DCA showed that the predictive nomogram provided the greatest net benefit compared with “no” or “all” (**Figure 7**). These results indicated that the use of the proposed nomogram to differentiate between MF-ICC and metastatic colorectal adenocarcinoma would provide a net benefit for almost all threshold probabilities in both the training and validation sets.

**Table 3:** The univariable and multivariable analysis of the logistic regression in diagnosing MF-ICC in the training sets.

Variables	Univariable factors	*P* value	Multivariable factors	*P* value
Clinical features	OR (95%CI)		OR (95%CI)	
**AFP (**≥20**ng/mL)**	2.31(0.75-7.07)	0.841		
**CA19-9(**≥36**U/mL)**	3.70(2.14-6.40)	<0.001	2.81(1.06-7.44)	0.037
**CEA(**≥5**ng/mL)**	0.19(0.11-0.33)	<0.001	0.05(0.02-0.16)	<0.001
**CA125 (**≥35**ng/mL)**	7.42(3.12-17.62)	<0.001	10.21 (2.54-40.99)	0.001
**Lesion size (**≥5**cm)**	9.53(5.10-17.80)	<0.001	13.99(4.82-40.68)	<0.001
**Lesion number(single)**	2.28(1.35-3.85)	0.002	5.58(2.09-14.91)	0.001
**Abnormal lymph node**	4.58(1.98-10.59)	0.018		
**Bile duct dilation**	5.23(1.38-19.82)	0.015		
**Echogenicity**				
hypo	reference	reference	reference	
iso	0.23(0.07-0.98)	0.048	0.15(0.018-1.34)	0.092
hyper	0.20(0.10-0.41)	<0.001	0.23(0.07-0.65)	0.007
**Irregular Shape**	2.56(1.44-4.54)	0.001	1.91(0.75-4.88)	0.173
**Rim APHE**	0.41(0.25-0.68)	<0.001	0.36(0.14- 0.92)	0.034
**Unclear boundary of intratumor non-enhanced area**				
yes	2.80(1.27-6.17)	0.01		
no	reference			
**Tumor necrosis**	2.79(1.57-4.95)	<0.001	3.78(1.37-10.38)	0.01
**Necrosis area**				
absence	reference	reference		
< 50% area	3.10(1.33-7.23)	0.009		
≥50% area	2.61(1.32-5.18)	0.006		

MF-ICC, mass-forming intrahepatic cholangiocarcinoma; OR, odds ratio; CI, confidence interval. AFP, alpha fetoprotein; CA19-9, carbohydrate antigen 19-9; CEA, carcinoembryonic antigen; CA125, carbohydrate antigen 125; APHE, arterial phase hyper- enhancement.

## 4 Discussion

ICC is the second most common primary hepatic malignancy accounting for 10% to 20% of newly diagnosed liver cancers (15). Distinguishing MF-ICC from liver metastatic colorectal adenocarcinoma is a challenging task due to their similar imaging features and lack of specific immunohistopathological biomarkers (16, 17). Accurate differentiation is crucial for appropriate intervention and better prognostic assessment, given the differences in treatment between the two entities. Through multivariate logistic regression analysis, elevated CA19-9 level (P=0.037), elevated CA125 level (P=0.001), lower CEA level (P<0.001), tumor size≥5cm (P<0.001), single lesion (P=0.001), hypo-echogenicity (P=0.007), tumor necrosis (P=0.01), and absence of rim APHE (P=0.034) were identified as independent factors for the diagnosis of MF-ICC. The developed nomogram based on clinical indexes and imaging characteristics of BMUS and CEUS achieved high accuracy in differentiating MF-ICC and metastatic colorectal adenocarcinoma, AUC value of 0.937 (95%CI: 0.907,0.969) and 0.916 (95%CI: 0.863,0.968) in training and validation sets, respectively.

The previous study by Conway et al. (17) found that MF-ICCs more frequently presented as a single large mass compared to metastasis nodules. The current study found similar results, with larger size and single lesion more commonly seen in MF-ICC than in metastasis nodules. Hypoechoic appearance was also more frequently found in MF-ICC group than metastasis group, which is consistent with previous investigations (18–20). However, the conventional ultrasound imaging findings for MF-ICC are not specific, as they can show diverse echo patterns and may be hypo-, iso-, or hyperechoic, and homogenous or heterogeneous (19, 21). The grayscale sonographic appearances of liver metastases are various. They can be hypoechoic or hyperechoic, but hyperechoic metastases are mainly of gastrointestinal origin (22, 23). The current study also found that hyperechoic appearance was more commonly identified in metastatic colorectal adenocarcinoma group than in MF-ICC group.

Although MF-ICC has been characterized by rim hyperenhancement during the arterial phase, it also can be seen in metastasis due to intralesional coagulative necrosis and perilesional, nontumoral areas with desmoplastic reaction, inflammatory cell infiltration, or vascular proliferation (24, 25). In the present study, rim APHE was observed more commonly in metastasis (55.8%) and was significantly different from MF-ICCs (34.9%). Inconsistently, the studies from Jin et al. (26) and Huang et al. (25) found that peritumoral enhancement were detected in 51.6% and 50.5% of patients with all MF-ICCs. This could be explained by larger size in their studies (median size 6.3 ± 2.8cm, 6.51 ± 3.08 cm, respectively), resulting in viable cells at the periphery of the tumor and rich edematous internal stroma. Furthermore, some researchers had described a more frequent rim-like hyperenhancement pattern of hypo-vascular metastases, due to low arterial perfusion (27). Consistently, our study also revealed that the rim APHE was the most common feature of metastasis colorectal adenocarcinoma. Furthermore, most lesions of MF-ICC show non-enhanced area inside the tumor more commonly than liver metastases. This characteristic was consistent with the prior studies by Chen et al, Min et al. and Tian et al. (19, 28, 29). One plausible reason might be that the larger MF-ICC had more fibrous tissues and necrosis inside the tumor.

Tumor markers play an important role in the management of many patients with cancer, because these values are readily available, inexpensive, and can be obtained preoperatively (30). CA19-9 is a blood tumor marker and was discovered in patients with colon cancer and pancreatic cancer in 1981 (31). Previously, serum levels of CA 19-9 have been reported to be useful for the diagnosis of ICC (32, 33). In our study, we found that elevated CA19-9 level was more frequently observed in MF-ICC group than metastatic group, which was parallel to those of previous studies. CA125 is primarily used for early diagnosis of pancreatic cancer and has differential value for some benign and malignant digestive tumors (34). Moreover, the studies by Higashi et al. (35) indicate that CA125 expression is a prognostic factor for poor survival in MF-ICC. In our study, elevated serum levels of CA-125 were more frequently observed in the MF-ICC group than in the metastatic group. This feature was further confirmed as a predictor by multivariable logistic analysis to identify MF-ICC from hepatic metastasis. CEA was first discovered in fetal gut tissue and gastrointestinal tract tumors many years ago, and subsequently detected in the circulation of patients, becoming a recognized serum marker for colorectal cancer. Furthermore, in patients with metastatic colorectal cancer, CEA has been reported as a prognostic factor for predicting recurrence and survival time (36). The liver is a common site for the spread of malignancy, with approximately 15%-25% of colorectal cancer patients having synchronous liver metastases and a similar portion developing liver metastases after colorectal resection (37). Consequently, we observed a higher incidence of elevated CEA levels in liver metastases than in MF-ICC, which is consistent with the findings of Nystrom et al. (38).

Previous studies have explored the potential of CEUS in distinguishing MF-ICC from other liver malignancies. However, there is limited research that focuses on using ultrasound features to differentiate MF-ICC from liver metastases. Our nomogram, which incorporates CEUS characteristics, tumor markers, and BMUS features, demonstrates strong discriminatory ability between MF-ICC and metastatic colorectal adenocarcinoma. Moreover, the inclusion of easily obtainable imaging features and clinical indicators from preoperative examination makes the nomogram highly applicable in clinical settings.

Despite the favorable performance of our nomogram, several limitations exist in our study. First, we used retrospective data from a single-center experience of patients, which has the potential for selection bias and limits the generalizability of our findings. Second, in multiple lesions, we chose the largest one for analysis. While this choice has its rationale in certain aspects, we must acknowledge the evidence limitations associated with this approach. For instance, this selection may overlook the potential significance of smaller lesions in differential diagnosis. Third, the lack of external validation is a significant limitation of our study. Without external validation, there is a risk of overestimating the model’s performance and limiting the understanding of its real -world applicability. Therefore, it is crucial to undertake additional multicenter prospective studies to validate the diagnostic ability of the nomogram.

## 5 Conclusion

In summary, our study identified several independent risk factors for MF-ICC, including single lesion, tumor size ≥5cm, hypo-echogenicity, presence of tumor necrosis, absence of rim APHE, lower CEA level, elevated CA19-9 and CA-125 level. Based on these characteristics, we developed a nomogram that can accurately and robustly differentiate MF-ICC from liver metastatic colorectal adenocarcinoma. This nomogram has potential as a valuable clinical tool for preoperative diagnosis and treatment selection. However, further multicenter prospective studies are needed to validate our findings and improve the clinical applicability of the nomogram.

## Data availability statement

The original contributions presented in the study are included in the article/**Supplementary Material**. Further inquiries can be directed to the corresponding author.

## Ethics statement

The studies involving humans were approved by Biomedical Ethics Review Committee of West China Hospital of Sichuan University. The ethics committee/institutional review board waived the requirement of written informed consent for participation.

## Author contributions

WB and ML prepared the study design and manuscript. JY and JH were responsible for statistical analysis. KZ supported the data acquisition and manuscript revision. QL supervised the writing and revision of the manuscript. All authors contributed to the article and approved the submitted version.
