# Supplementary material for: A nomogram based on ultrasonographic features and clinical indicators for differentiating mass-forming intrahepatic cholangiocarcinoma and liver metastatic colorectal adenocarcinoma
Source: Front Oncol. 2023 Oct 31;13:1245686. doi: 10.3389/fonc.2023.1245686 (PMC10644673; doi:10.3389/fonc.2023.1245686)
Supplement: Supplementary file 1 [file Table_1.docx]

Supplementary Material

# Supplementary table:

Supplementary Table S1 Inter-reader agreement of major BMUS and CEUS features.

| **Variables** | | **reviewer 1** | | **reviewer 2** | | κ* | |
| --- | --- | --- | --- | --- | --- | --- | --- |
| **BMUS** | |  | |  | |  | |
| **Echogenicity(hypo/iso/hyper)** | | 250/14/79 | | 244/15/84 | | 0.526 | |
| **Irregular shape** | | 277 | | 185 | | 0.095 | |
| **Ill- defined boundary** | | 299 | | 202 | | 0.1 | |
| ***CEUS*** | |  | |  | |  | |
| **Rim APHE** | | 198 | | 116 | | 0.573 | |
| **Early washout** | | 127 | | 189 | | 0.464 | |
| **Marked washout** | | 54 | | 42 | | 0.504 | |
| **Unclear boundary of intratumor non-enhanced area** | | 57 | | 81 | | 0.253 | |
| **Tumor necrosis** | | 273 | | 294 | | 0.472 | |
| **Necrosis area (**absent/< 50%/≥50%**)** | | 232/46/63 | | 249/46/48 | | 0.431 | |

BMUS: B- mode ultrasound; CEUS: contrast enhanced ultrasound; MF-ICC: mass-forming intrahepatic cholangiocarcinoma; APHE: arterial phase hyper- enhancement; AP: arterial phase; PP: portal phase; LL: late phase; Data are number of nodules; *κ values 0.81–1.0, 0.61–0.80, 0.41–0.60, 0.21–0.40, and 0.00–0.20 considered to be almost perfect, substantial, moderate, fair, and slight, respectively.

**
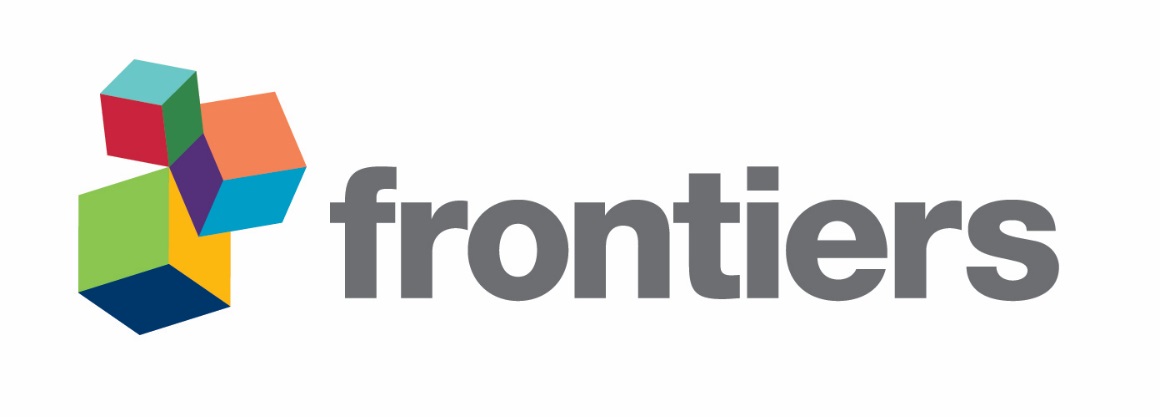
**
